# Community Based Management of COVID-19 as a Way Forward for Pandemic Response

**DOI:** 10.3389/fpubh.2020.589772

**Published:** 2021-01-13

**Authors:** Sunil Kumar Panigrahi, Sagarika Majumdar, Abhiruchi Galhotra, Santosh Chanbasappa Kadle, Ashis Samuel John

**Affiliations:** ^1^Department of Community and Family Medicine, All India Institute of Medical Sciences, Raipur, India; ^2^Department of Obstetrics and Gynecology, All India Institute of Medical Sciences, Raipur, India; ^3^District Health Services, Jalna, India; ^4^Regional Technical Research Centre for Health Technology Assessment, Sree Chitra Tirunal Institute for Medical Sciences and Technology, Thiruvananthapuram, India

**Keywords:** COVID-19, community based management, health system, capacity, pandemic response

## Introduction

The COVID-19 pandemic, which began with reports of atypical pneumonia cases in the city of Wuhan in Hubei province of China, was declared a public health emergency of international significance by the WHO on January, 30 2020. The virus SARS COV2 has, to date, infected more than 16 million people around the world and over 0.6 million people have succumbed to it. The pandemic has affected more than 220 countries (1). Some of the most developed economies, with state of art healthcare systems and advanced diagnostic and critical care facilities, have been worst affected by the pandemic (2). Deaths among COVID-19 cases are not only because of the pathogenesis of the virus but also because of increases in patients, which overwhelms healthcare institutions with unexpected surges in cases (3). Low and middle-income countries in South East Asia and Africa with inadequate health capacity, may face dire consequences after a surge of cases and loss of lives. Many international health agencies including the WHO have advised that “flattening the curve” and strengthening healthcare capacity can reduce the chances of overwhelming health systems (4).

The response in India, a LMIC, has focused on interrupting the transmission of infection through contact tracing, finding and isolating cases when they occur. The guidelines of the Ministry of Health recommends COVID care centers (CCC) be used for mild or very mild cases, separate dedicated COVID health centers (DCHC) for moderate cases (pneumonia without any signs of severe disease), and dedicated COVID hospitals with ICU facility (DCH) for critical cases (5).

## Need for Community Based Management

Though the steps taken in terms of prevention, interruption of transmission, and management of COVID-19 cases in India have been proactive, there is an urgent need to strengthen and integrate COVID care into the primary health care of the country. There exists a huge disparity between healthcare capacities among different states in India and states do not have similar capacities to and the ability to provide enough stand-alone centers like CCCs, DCHCs, or DCHs (6). Even in developed states in India, the rural-urban disparity is huge. Most often multi-specialty hospitals are centered on cities. The response against a prolonged and protracted pandemic with possible multiple waves of infection in the future will require more sustainable COVID-19 management through primary health care approaches and community participation instead of a vertical system of policy planning and management (7).

## Planning and Implementation of Community-Based Management of COVID-19

We propose a three-tier system of community-based management of COVID-19 with a primary health care approach:

- Tier I (Center for public health planning and implementation): Local Self Government (Village level Panchayatiraj Institutions/ Urban Local bodies)
- Tier II (Center of medical management of COVID-19 in the community): CHC (Community Health Center or the first referral center)
- Tier III (Center of IEC planning, Surveillance, and Inter-sectoral/ Inter-agency coordination): District.

## Discussion

The current practice in India focuses on the identification of suspects and confirmation of cases through RT-PCR testing. The ministry of health recommends the early segregation of cases into mild, moderate, and severe categories. The imperfect execution of segregation in many places has resulted in 77% of all patients admitted to hospitals having only mild symptoms of COVID-19 (8). Moreover, mild disease may worsen into a moderate or severe category. A dedicated referral channel is a necessity during the surge of cases. Due to the absence of fore-referral confirmation of the availability of ICU beds in the referral facility, there have been instances of denial or unavailability of critical care after the patient has reached the hospital (9).

We suggest that cases should be segregated based on the probability of future complications. Older patients with co-morbidities, pregnant mothers, children under 5 years of age, and patients exposed to high viral load in large closed door congregations should be admitted to dedicated COVID-19 hospitals with ICU facilities (10–12). Patients with a lesser probability of complications can be managed in village-level isolation centers if they are asymptomatic and Community Health Centers (COVID CHCs) if symptomatic. Village level isolation centers will not only help to enhance community engagement and improve risk communication but also promote local innovations for infection control through community participation, which are essential for a sustained and successful pandemic response (13–16). Some states have tried to decentralize decision making with success during the ongoing COVID-19 pandemic (17, 18).

The current practice of taking diagnosed COVID-19 patients to standalone healthcare centers promotes over-reliance on the health system, as a suspect is isolated from the community from the time of its contact tracing and quarantine through to testing, diagnosis, management, and finally, discharge (average 30 days) from the hospital. This increases the fear of disease among the population, which in turn increases the stigma not only against patients but also their family members, the recovered patients, and the health care workers who continue to stay in the community (19, 20). This makes contact tracing and the self-declaration of travel history or gathering exposure history difficult. Village level isolation centers will ensure that the management and recovery of asymptomatic and mild cases takes place under direct community supervision, with technical help from mobile medical units (Table 1), addressing the myriad social issues and stigma associated with COVID-19.

**Table 1:**
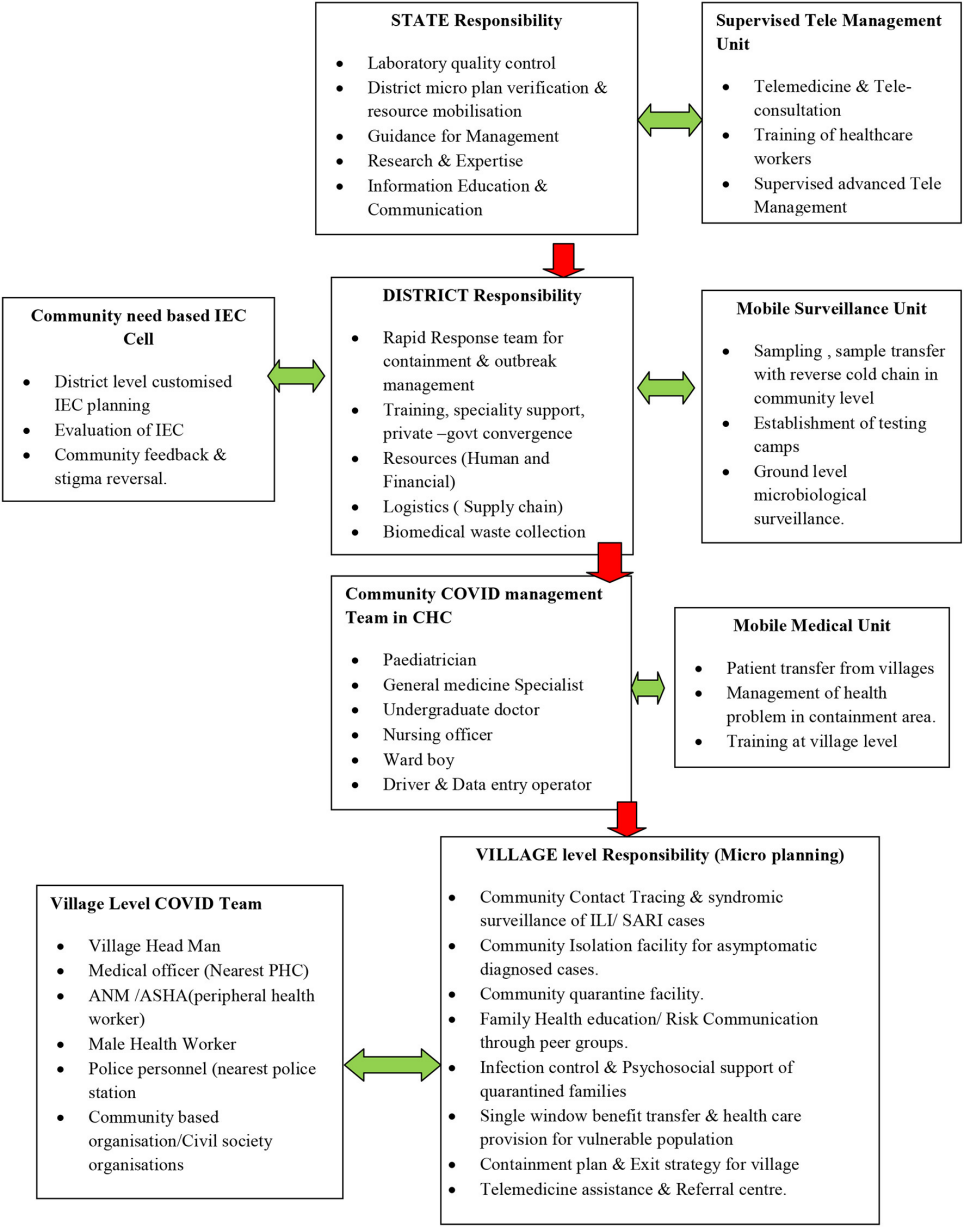
Flowchart of health system structure for the community-based management of COVID-19 cases.

**Figure 1:**
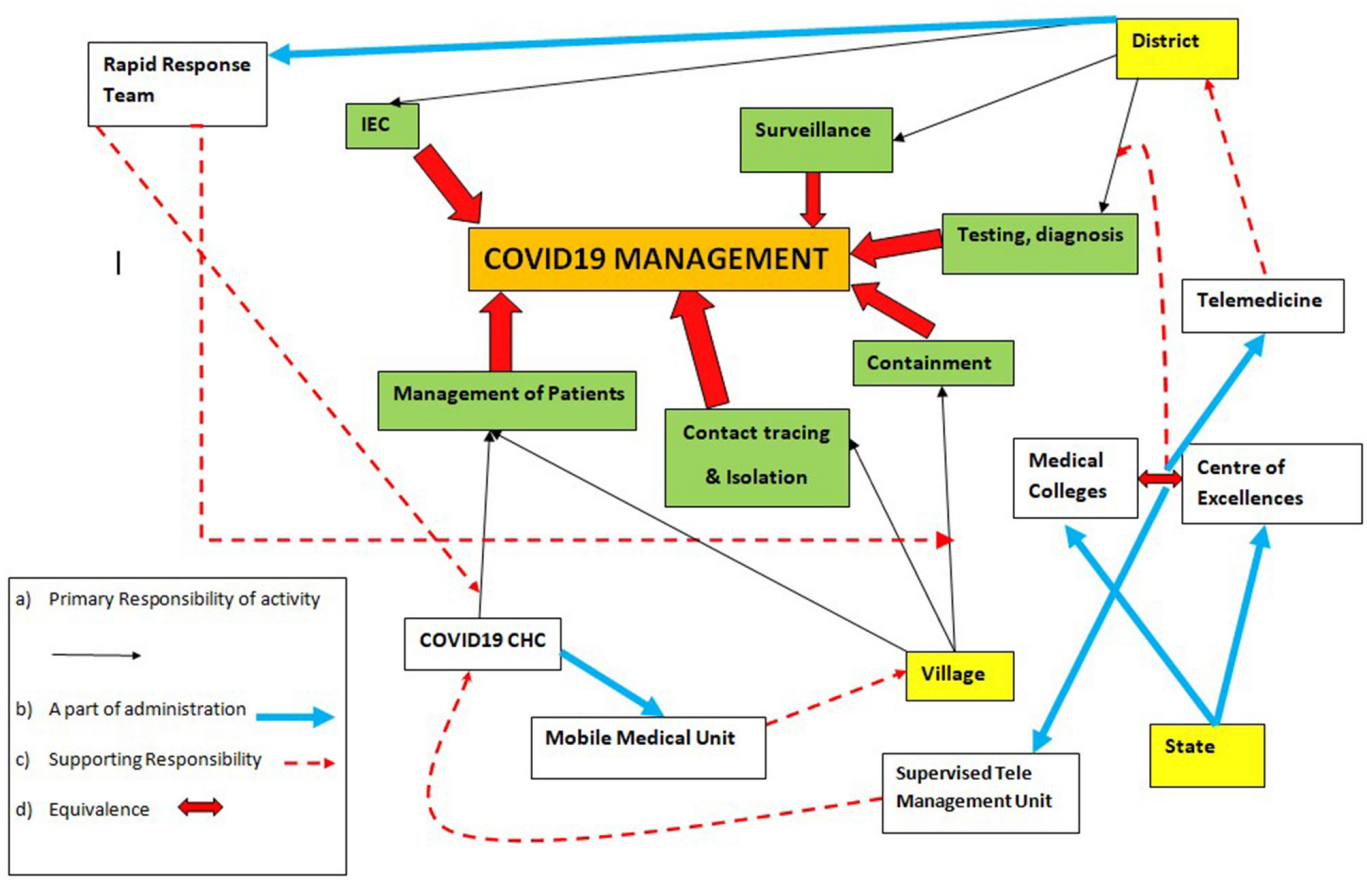
Flowchart for operational health system for community based management of COVID-19.

The Involvement of Community Health Centers (CHCs) the management of COVID19 will help diversify and decentralize the health care system in rural and semi-urban areas (Figure 1). It could lead to vital skill improvement in the management of infectious disease among health care workers including doctors in CHCs through supervised management of cases from medical colleges and centers of excellence. Supervised management through advanced telemedicine as a potential way of healthcare delivery for COVID-19 management is being increasingly used by some states in India with success (21). The involvement of at least 40% of CHCs all over the country could help create over 75,000 beds for mild to moderately sick COVID patients. This will help create a specialist pool of trainers, doctors, nurses, and infection control personnel who are equipped to handle the spread of infectious disease spread in the long term and possible future waves of the COVID-19 pandemic in the community (22, 23). This will spare the rest of the health care system and help ensure the continuation of essential and emergency health services by the other 60% CHCs, Sub-district, and District hospitals. This will help in integrating COVID-19 response into the primary health care system of the country.

One of the most important bottlenecks in the pandemic has been the lack of standardized, community-oriented, inclusive information, and a cohesive education and communication (IEC) strategy. A specialized IEC cell, with a community feedback mechanism under the district administration (Table 1) will help create community need-based, culturally appropriate IECs. This will improve the engagement of different communities by improving awareness, reducing prevailing stigma, and countering misinformation about the disease.

The current practice of laboratory diagnosis starts with the collection of nasopharyngeal and/or oro-pharyngeal swab by laboratory personnel or healthcare workers in individual hospitals or quarantine facilities or isolation centers. The samples are then sent to the designated laboratories for rt-PCR by maintaining the reverse cold chain (24). This increases the number of healthcare staff exposed to an aerosol-generating procedure of sample collection which leads to higher consumption of full COVID PPE. A specialized mobile surveillance unit at the district level can be mobilized for all facility-based and community-based sample collection under district administration. This will ensure quality sample collection by a specialized team which can help streamline laboratory diagnosis through better reverse cold chain maintenance.

The pandemic provides an opportune moment to expand primary healthcare infrastructure along with a once redundant network of telemedicine at community levels in India by resurrecting village-level resource centers (25).

## Conclusion

The community-based management of COVID-19 cases will not only help in reducing the fear and stigma prevailing during this pandemic but also enhance community engagement through participation and an increase in healthcare capacity. This will also improve community preparedness for future re-emergence of infectious diseases and will reduce the need for drastic measures like lockdowns in the future.

## Author Contributions

SP: concept, intellectual content, and manuscript editing. SM: intellectual content and manuscript preparation. AJ: literature search and manuscript preparation. SK: literature search and manuscript preparation. AG: intellectual content and literature search. All authors contributed to the article and approved the submitted version.

## Conflict of Interest

The authors declare that the research was conducted in the absence of any commercial or financial relationships that could be construed as a potential conflict of interest.

## References

[1] Coronavirus Disease 2019. World Health Organisation (2020). Available online at: https://www.who.int/emergencies/diseases/novel-coronavirus-2019 (accessed May 3, 2020).

[2] Mortality Analyses—Johns Hopkins Coronavirus Resource Center Available online at: https://coronavirus.jhu.edu/data/mortality (accessed May 3, 2020).

[3] Cavallo JJ Donoho DA Forman HP Hospital capacity and operations in the coronavirus disease 2019 (COVID-19) pandemic—planning for the Nth patient. JAMA Heal Forum. (2020) 1:e200345 10.1001/JAMAHEALTHFORUM.2020.0345 36218595

[4] EXPLAINER: “Flattening the curve” Available online at: www.who.int (accessed May 3, 2020).

[5] Guidance Document on Appropriate Management of Suspect/Confirmed Cases of COVID-19 New Delhi: Ministry of Health and Family Welfare, (2020) p. 7 Available online at: https://www.mohfw.gov.in/pdf/FinalGuidanceonMangaementofCovidcasesversion2.pdf

[6] COVID-19 in India: State-Wise Estimates of Current Hospital Beds ICU Beds and Ventilators Center for Disease Dynamics, Economics and Policy. Available online at: https://cddep.org/publications/covid-19-in-india-state-wise-estimates-of-current-hospital-beds-icu-beds-and-ventilators/ (accessed May 6, 2020).

[7] Report on Healthcare Access Initiatives. (2016). Available online at: https://www.indiaoppi.com/wp-content/uploads/2019/12/Report-on-healthcare-access-initiatives-For-web.pdf (accessed May 8, 2020)

[8] Report of COVID-19 Cases 29 April 2020 Mumbai: Public Health Department, Government of Maharashtra (2020) p. 18.

[9] Mumbai: 36-Year-Old Doctor Dies of Multiple Organ Failure Samples Test Positive For Covid-19—India News Available online at: https://www.indiatoday.in/india/story/mumbai-36-year-old-doctor-dies-of-multiple-organ-failure-samples-test-positive-for-covid-19-1670764-2020-04-24 (accessed May 7, 2020).

[10] Zhou F Yu T Du R Fan G Liu Y Liu Z Clinical course and risk factors for mortality of adult inpatients with COVID-19 in Wuhan, China: a retrospective cohort study. Lancet. (2020) 395:1054–62. 10.1016/S0140-6736(20)30566-3 32171076 PMC7270627

[11] Wu Z McGoogan JM. Characteristics of and important lessons from the coronavirus disease 2019 (COVID-19) outbreak in China: summary of a report of 72314 cases from the Chinese Center for Disease Control and Prevention. JAMA. (2020) 323:1239–42. 10.1001/jama.2020.2648 32091533

[12] Huang C Wang Y Li X Ren L Zhao J Hu Y. Clinical features of patients infected with 2019 novel coronavirus in Wuhan, China. Lancet. (2020) 395:497–506. 10.1016/S0140-6736(20)30183-5 31986264 PMC7159299

[13] Marston C Renedo A Miles S. Community participation is crucial in a pandemic. Lancet. (2020) 395:1676–78. 10.1016/S0140-6736(20)31054-0 32380042 PMC7198202

[14] Risk Communication and Community Engagement (RCCE) Readiness and Response to the 2019 Novel Coronavirus (2019-nCoV) Available online at: https://www.who.int/publications-detail/risk-communication-and-community-engagement-readiness-and-initial-response-for-novel-coronaviruses-(-ncov) (accessed May 7, 2020).

[15] Community-Based Health Care Including Outreach and Campaigns in the Context of the COVID-19 Pandemic (2020).

[16] Risk Communication and Community Engagement (RCCE) Action Plan Guidance COVID-19 Preparedness and Response Geneva: World Health Organisation (2020) p. 26 Available online at: https://www.who.int/publications-detail/risk-communication-and-community-engagement-(rcce)-action-plan-guidance (accessed May 7, 2020).

[17] The Panchayati Front: Tap Potential of Local Self-Government to Fight COVID-19 The Indian Express. Available online at: https://indianexpress.com/article/opinion/columns/coronavirus-pandemic-panchayati-front-6395715/ (accessed May 7, 2020).

[18] What Nation can Learn from Kerala: Lockdown is Not Enough. Preparedness, Decentralisation, are Key. The Indian Express. Available online at: https://indianexpress.com/article/opinion/columns/coronavirus-covid-19-kerala-curve-6365935/ (accessed May 7, 2020).

[19] Ghaziabad Housing Society Bans Entry of Resident Doctors Out of COVID-19 Fears; AIIMS RDA Pens Letter to Amit Shah DNA (2020). Available online at: https://www.dnaindia.com/india/report-ghaziabad-housing-society-bans-entry-of-resident-doctors-out-of-covid-19-fears-aiims-rda-pens-letter-to-amit-shah-2824004 (accessed May 8, 2020).

[20] Recovered Coronavirus Patients Face Social Stigma in Bihar. Times of India (2020). Available online at: https://timesofindia.indiatimes.com/city/patna/recovered-corona-patients-face-social-stigma-in-state/articleshow/75071886.cms (accessed May 8, 2020).

[21] Crack Team of Nine Doctors Treats City's Most Critical Cases Available online at: https://mumbaimirror.indiatimes.com/coronavirus/news/crack-team-of-nine-doctors-treats-citys-most-critical-cases/articleshow/75524743.cms (accessed May 8, 2020).

[22] How India Must Prepare for a Second Wave of COVID-19—Nature India Available online at: https://www.natureasia.com/en/nindia/article/10.1038/nindia.2020.80 (accessed May 8, 2020).

[23] Xu S Li Y. Beware of the second wave of COVID-19. Lancet. (2020) 395:321–2. 10.1016/S0140-6736(20)30845-X 32277876 PMC7194658

[24] Specimen Collection Packaging and Transport Guidelines for 2019 Novel Coronavirus (2019-nCoV) (2020). Available online at: www.mohfw.gov.in (accessed May 8, 2020).

[25] Chellaiyan V Nirupama A Taneja N. Telemedicine in India: Where do we stand? J Fam Med Prim Care. (2019) 8:1872. 10.4103/jfmpc.jfmpc_264_19 31334148 PMC6618173

